# Tranexamic acid in total shoulder arthroplasty and reverse shoulder arthroplasty: a systematic review and meta-analysis

**DOI:** 10.1186/s12891-018-1972-3

**Published:** 2018-02-17

**Authors:** Liang-Tseng Kuo, Wei-Hsiu Hsu, Ching-Chi Chi, Jae Chul Yoo

**Affiliations:** 1 Division of Sports Medicine, Department of Orthopedic Surgery, Chang Gung Memorial Hospital, Chiayi, Taiwan; 2 Centre for Evidence-Based Medicine, Chang Gung Memorial Hospital, Chiayi, Taiwan; 3 Chang Gung University of Science and Technology, Chiayi, Taiwan; 4 College of Medicine, Chang Gung University, Taoyuan, Taiwan; 5 Department of Dermatology, Chang Gung Memorial Hospital, Linkou, 5, Fuxing St, Guishan Dist, Taoyuan, 33305 Taiwan; 6 Department of Orthopedic Surgery, College of Medicine, Samsung Medical Center, Sungkyunkwan University School of Medicine, 81 Irwon-ro, Gangnam-gu, Seoul, 135-710 Republic of Korea

**Keywords:** Total shoulder arthroplasty, Reverse total shoulder arthroplasty, Tranexamic acid, Blood loss, Transfusion

## Abstract

**Background:**

The effects of tranexamic acid (TXA) in the setting of shoulder arthroplasty are unclear. The objective of this study was to examine the effects of TXA in reducing the need for blood transfusions and blood loss in patients undergoing primary total shoulder arthroplasty (TSA) and reverse total shoulder arthroplasty (RTSA).

**Methods:**

We conducted a systematic review and meta-analysis of randomized controlled trials (RCTs) and retrospective cohort studies (RCS) that compared outcomes of patients who did and did not receive TXA during TSA or RTSA. We searched Cochrane Central Register of Controlled Trials, EMBASE, and MEDLINE for relevant studies. We assessed the risk of bias of the included studies and calculated pooled risk estimates. The primary outcome was transfusion rate, and secondary outcomes were changes in hemoglobin, estimated total blood loss (ETBL), blood loss via drainage, operative time, hospital stay, overall complications, and thromboembolic events.

**Results:**

We identified 3 RCTs and 3 RCS including 677 patients with 680 shoulders (343 TXA and 337 non-TXA). The random-effects model meta-analysis showed that TXA group had a lower transfusion rate (risk ratio (RR) 0.34, 95% CI 0.14 to 0.79), less change in hemoglobin (mean difference (MD) -0.64 g/dl, 95% CI -0.81 to − 0.46), and reduced ETBL (MD -249.24 ml, 95% CI -338.74 to − 159.74). In patients with RTSA, the TXA group had a lower transfusion rate (RR 0.28, 95% CI 0.14 to 0.79), less ETBL (MD -249.15 ml, 95% CI -426.60 to − 71.70), less change in hemoglobin (MD − 0.64 g/dl, 95% CI -0.86 to − 0.42), and less blood loss via drainage (MD − 84.56 ml, 95% CI -145.72.14 to − 23.39) than non-TXA group.

**Conclusions:**

The use of TXA in primary shoulder arthroplasty appears safe, and can reduce transfusion rate, changes in hemoglobin, and perioperative total blood loss, especially in patients with RTSA.

Level of Evidence: Systematic Review and meta-analysis, III.

**Electronic supplementary material:**

The online version of this article (10.1186/s12891-018-1972-3) contains supplementary material, which is available to authorized users.

## Background

With improvements in implant design and surgical techniques, total shoulder arthroplasty (TSA) and reverse total shoulder arthroplasty (RTSA) are gaining popularity and are widely indicated for various shoulder conditions including end-stage shoulder arthropathy, cuff tear arthropathy, traumatic shoulder injuries, tumors, and failure of prior arthroplasty. However, shoulder arthroplasty, including both TSA and RTSA, is associated with a considerable risk of perioperative blood loss, and a reported allogeneic blood transfusion rate ranging from 4.3% to 43% [1–7]. The risk factors for requiring a transfusion after shoulder arthroplasty include old age, female sex, preoperative anemia, ischemic heart disease, and reverse shoulder replacement [2–4, 6–8].

The complications of blood transfusions include allergic reactions, immunosuppression, infection, and transfusion-related cardiopulmonary injury [9, 10]. A previous study of a healthcare database reported that patients who received a perioperative blood transfusion had a higher risk of medical complications including myocardial infarction, pneumonia, sepsis, and cerebrovascular accidents, as well as venous thromboembolic events and surgical complications including periprosthetic infections, periprosthetic fractures, and mechanical complications [11]. Though sicker patients that are more likely to require transfusions are also more likely to have complications mentioned above such as MI, pneumonia, stroke, etc. It is not likely the transfusion itself that causes these complications.

Many methods are available to reduce perioperative blood loss, including hypotensive anesthesia, hemodilution, autologous blood transfusion, reinfusion drainage, and the use of intravenous or topical tranexamic acid (TXA) [12, 13]. TXA interferes with the process of fibrinolysis and thereby reduces perioperative blood loss and the need for a transfusion [14]. Previous systematic reviews and meta-analyses have shown that the use of TXA in total knee arthroplasty and total hip arthroplasty can reduce blood loss and blood transfusion rates without increasing venous thromboembolism or other complications [15–18]. However, even though a few studies have investigated the use of TXA in shoulder arthroplasty, its effectiveness remains unclear [19, 20]. To date, few meta-analysis of randomized controlled trials (RCTs) and retrospective cohort studies (RCS) has investigated the effects of TXA in TSA [21, 22]. Even though more current studies on this issue have been published recently [23, 24], systematic evaluations of the latest evidence on the use of TXA in shoulder arthroplasty, and especially in RTSA, are lacking. Therefore, to comprehensively examine the effects of TXA in shoulder arthroplasty, we conducted this systematic review and meta-analysis to evaluate outcomes including transfusion rate, total blood loss, changes in postoperative hemoglobin (Hb), operative time, hospital stay and thromboembolic events. Our hypothesis is that TXA can reduce allogenic blood transfusion rate and blood loss effectively in patients with shoulder arthroplasty.

## Methods

### Data source and search strategy

We searched the Cochrane Central Register of Controlled Trials (CENTRAL), EMBASE, and MEDLINE from inception to August 15th, 2017 for RCTs and RCS that compared surgical outcomes in patients who did and did not receive TXA during TSA or RTSA. We also checked the references of the included studies for potentially relevant studies. There were no language restrictions.

The key words used in the search included “tranexamic acid”, “total shoulder arthroplasty”, and “reverse total shoulder arthroplasty”. Search details are shown in the supplementary document (see Additional file 1: Appendix 1). We also searched the U.S. National Institutes of Health trials registry (http://clinicaltrials.gov). In addition, we contacted experts in this field for relevant ongoing trials or unpublished studies.

### Selection criteria

Studies were included if they met the following criteria: (1) they were designed as a RCT or RCS; and (2) they compared the outcomes in patients who did and did not receive TXA during TSA or RTSA. There were no restrictions on the route of TXA administration. Two authors (LTK and CCC) independently checked the citations identified from the searches against the inclusion criteria.

### Data extraction and risk of bias assessment

Two authors (LTK and WHH) independently extracted the data from the included trials using a formal data extraction sheet. The items were as follows: first author, year of publication, diagnosis, study design, sample size, participant characteristics (e.g., age and sex), the regimen of TXA (dose and route), and the operative details such as the prosthesis type and surgical approach. Outcome data including operative time (minutes), hospital stay (days), blood loss through an intra-articular drain (ml), estimated blood loss (ml), changes in postoperative Hb (g/dl), transfusion rate and complications including overall and thromboembolic events were also extracted. The third author (CCC) arbitrated in cases where LTK and WHH could not agree.

LTK and WHH independently assessed the risk of bias of the included studies, and CCC resolved differences in opinions. The Cochrane Collaboration’s tool was used to evaluate the risk of bias of the included RCTs. The Cochrane risk of bias tool included the following domains on biased estimates of intervention effects: randomization sequence, allocation concealment, performance bias (blinding of patients and personnel), detection bias (blinding of outcome assessors), attrition bias (incomplete outcome data), selective reporting, and other biases [25, 26]. For each domain, a high, low, or unclear risk of bias was judged per the quality of the RCT [25]. The Newcastle-Ottawa Scale was applied to assess the bias of the included RCS [27].

The primary outcome was blood transfusion rate. The secondary outcomes included estimated total blood loss, changes in postoperative Hb (g/dl), blood loss via the drain (ml), operative time (minutes), hospital stay (days), and thromboembolic and overall complications.

### Statistical analysis

Quantitative analysis was performed for blood loss via drainage, estimated total blood loss, changes in postoperative Hb, operative time, and hospital stay, for which continuous data were presented as mean difference (MD) with a 95% confidence interval (CI). Dichotomous data including transfusion rate, overall complications, and thrombotic complications were reported as risk ratio (RR) with a 95% CI. We examined between-study variance using the tau-square (τ 2) statistic [26]. χ^2^ and *I*^2^ statistics were used for statistical heterogeneity, and significance was set at *P* <  0.10. *I*^2^ values of 0–24.9%, 25–49.9%, 50–74.9%, and 75–100% were assigned as none, low, moderate, and high heterogeneity, respectively [28, 29]. We performed a random-effects model meta-analysis for all outcomes, because we expected clinical heterogeneity across the included RCTs and RCS [30]. For continuous data, if the standard deviation (SD) was not reported, we estimated the mean and variance from the reported median, range, and sample size as previously reported [31]. When the SD and range were not available, variance was estimated from the *P* value in the *t* test [26]. A forest plot was used to summarize the results. Review Manager 5.3 (The Nordic Cochrane Centre, The Cochrane Collaboration, 2014) was used for meta-analysis.

### Subgroup analysis

If data were available, we planned to perform subgroups analysis including:

1. RCTs alone;
2. Different routes of TXA administration: intravenous (IV) versus topical;
3. RTSA or TSA only.

## Results

**Fig. 1:**
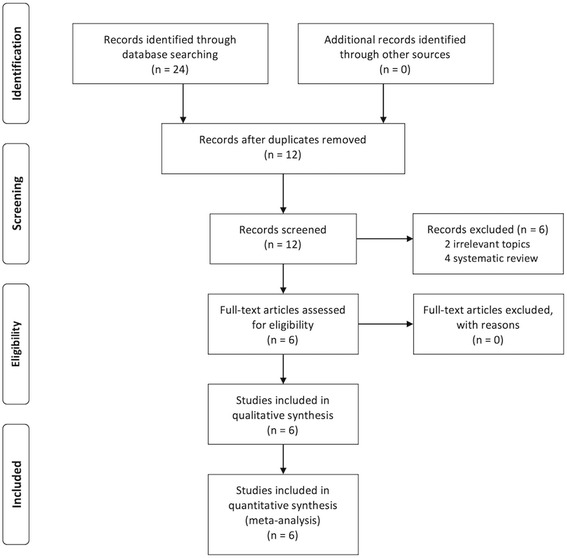
PRISMA 2009 (Preferred Reporting Items for Systematic Reviews and Meta-Analyses) flow diagram of the study

The details of the study selection process are presented in Fig. 1 [32]. Our search of the MEDLINE, EMBASE, and CENTRAL databases returned 24 published studies. No additional studies were identified from the references of the included studies. After excluding 12 duplicates and 6 studies (two irrelevant topics and four systematic reviews), we finally included the following six studies in this meta-analysis: Abildgaard et al. [33], Friedman et al. [19], Gillespie et al. [20], Kim et al. [24], Pauzenberger et al. [23], and Vara et al. [34].

### Study characteristics and patient populations

The included trials were published between 2015 and 2017 (Table 1). The sample sizes ranged from 48 to 194, with a total 677 patients (680 shoulders, 343 in the TXA group and 337 in the non-TXA group). Three included studies [20, 23, 34] were RCTs that prospectively compared outcomes, and the other three RCS [19, 24, 33] compared results using retrospective analysis (Table 1). Further perioperative characteristics of the included studies are described in Table 2.

**Table 1:** Characteristics of the included studies

Author	Year	Patients’ diagnosis	Surgery	Study design	Enrolled sample number (M/F)	Average age, years	Outcome measurement	Quality assessment
Gillespie [20]	2015	OA	TSA 44 RTSA 67	Randomized controlled trial	TXA 56 (27/29) Placebo 55 (22/33)	TXA: 67.59 Placebo:66.45	Blood loss via drainage, Hb change, ETBL, transfusions, complications	Detail shown in Fig. 2
Pauzenberger [20]	2017	Not mentioned	TSA 26 RTSA 28	Randomized controlled trial	TXA 27(20/7) Placebo 27(18/9)	TXA: 70.3 ± 9.3 Placebo:71.3 ± 7.9	Blood loss via drainage, Hb/Hct change, ETBL, haematoma, complications	Detail shown in Fig. 2
Vara [34]	2017	Massive rotator cuff deficiency ± glenohumeral arthrosis	RTSA 102	Randomized controlled trial	TXA 53(20/33) Placebo 49(22/27)	TXA: 67 ± 9 Placebo:66 ± 9	Blood loss via drainage, Hb change, ETBL, operation time, hospital stay, transfusions, complications	Detail shown in Fig. 2
Abildgaard [33]	2016	TSA:1 ON and 7 OA RTSA:45 CTA, 34 massive cuff tear, 1 ON, 1RA, 11OA, and 2 fractures	TSA 77 RTSA 94	Retrospective cohort study	TXA 77 (49/28) Placebo 94 (51/43)	TSA: TXA: 70 (53–87) Placebo:71 (58–87) RTSA TXA: 74 (54–90) Placebo: 76(54–89)	Blood loss via drainage, Hb/Hct change, ETBL, transfusions, complications	9^a^
Friedman [19]	2016	Not mentioned	TSA 97 RTSA 97	Retrospective cohort study	TXA 106 (46/60) Placebo 88 (33/55)	not mentioned	Hb/Hct, recover room time, operation time, hospital stay, transfusions, complications	8^a^
Kim [24]	2017	CTA	RTSA 48	Retrospective cohort study	TXA 24 (3/21) Placebo 24 (6/18)	TXA: 73.2 ± 4.4 Placebo: 74.2 ± 4.4	Hb/Hct change, operation time, blood loss via drainage	8^a^

*CTA* Cuff tear arthropathy; *ETBL* Estimated total blood loss; *F* Female; *Hb* Hemoglobin; *Hct* Hematocrit; *M* Male; *OA* Osteoarthritis; *ON* Osteonecrosis; *RA* Rheumatoid arthritis; *RTSA* Reverse total shoulder arthroplasty; *TSA* Total shoulder arthroplasty; *TXA* Tranexamic acid

^a^Quality scores derived from the Newcastle-Ottawa Scale

**Table 2:** Perioperative details of the included studies

Author	Year	Intervention	Prosthesis properties	Approach	Transfusion protocol	Thromboprophylaxis
Gillespie [20]	2015	TXA: 2 g in 100 ml NS for 5 min Placebo: 100 ml NS for 5 min	N/S	DP	1) Hb < 7.0 g/dl 2) 7.1 g/dl < Hb < 9.0 g/dl + symptoms	N/S
Pauzenberger [23]	2017	TXA: 1 g TXA intravenously in 100 ml NS, 2 doses Placebo: 100 ml NS, 2 doses	TSA (Eclipse; Arthrex Inc., Naples, Florida) RTSA (Delta Xtend; DePuy Synthes, Warsaw, Indiana)	DP	1) Hb < 8 g/dl 2) 8 g/dl < Hb < 10 g/dl + symptoms	Chemical prophylaxis (subcutaneous 40 mg of enoxaparin sodium + aspirin)
Vara [34]	2017	TXA: 10 mg/kg IV, 1st dose within 60 mins before surgery, 2nd dose at wound closure Placebo: NS	102 Non-cemented RTSA (79 Zimmer; 11 DePuy; 4 Biomet; 2 Encore,)	DP	1) Hb < 7 g/dl 2) 7 g/dl < Hb < 9 g/dl + symptoms	Chemical prophylaxis (subcutaneous heparin + oral aspirin) Mechanical prophylaxis (compression stockings)
Abildgaard [33]	2016	TXA: 1 g IV at skin preparation Placebo: no TXA	TSA (Bigliani/ Flatow Anatomical Total Shoulder; Zimmer, Warsaw, IN, USA) RTSA (Trabecular Metal Inverse/Reverse Total Shoulder, Zimmer)	DP	1) Hb < 7 g/dl 2) 7 g/dl < Hb < 9 g/dl + symptoms	Chemical prophylaxis was not routinely used postoperatively
Friedman [19]	2016	TXA: 20 mg/kg IV at skin preparation Placebo: no TXA	RTSA: cemented; brand N/S TSA: 90% non-cemented;	N/S	N/S	N/S
Kim [24]	2017	TXA: 500 mg IV	25 DJO Reverse Shoulder Prosthesis; 16 Tornier reverse shoulder prosthesis; 7 Biomet Comprehensive Reverse Total Shoulder Replacement	DP	No absolute guideline	N/S

*DP* Deltopectoral approach; *Hb* Hemoglobin; *IV* Intravenous; *NS* Normal saline; *N/S* Not shown; *RTSA* Reverse total shoulder arthroplasty; *TSA* Total shoulder arthroplasty; *TXA* Tranexamic acid

### Risk of bias of the included studies

**Fig. 2:**
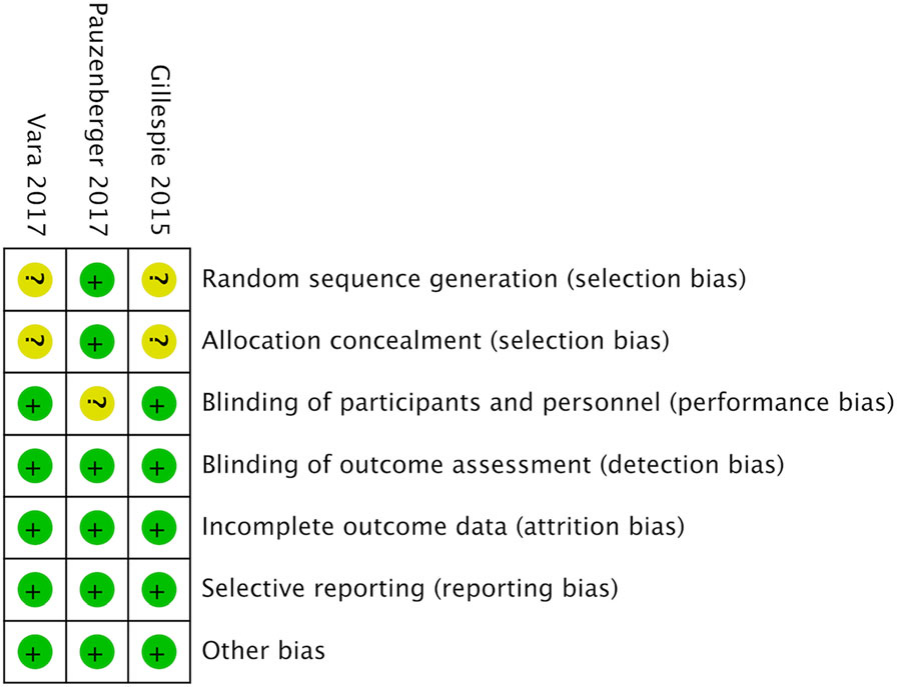
Risk of bias summary. Authors’ judgments about the risk of each bias item for each included study. “+” represents low risk of bias; “?” represents unclear risk of bias; “−” represents high risk of bias

Data on the risk of bias of the six studies included in the meta-analysis are summarized in Table 1 and Fig. 2. The three RCS [19, 24, 33] were of high quality (Newcastle-Ottawa Scale score > 7). For the risk of bias of the RCTs, random sequence generation and allocation concealment were not described in two trials [20, 34], and therefore the risk of bias was rated as being unclear. Blinding of the surgeon was not explicitly mentioned in one trial [23], which was therefore also rated as having an unclear risk of bias. Other items with regards to the risk of bias were rated as low risk when appraised using the Cochrane Collaboration’s tool.

### Outcomes

Summaries of the findings and subgroup analysis are shown in Table 3.

**Table 3:** Summary of findings of shoulder arthroplasty

Outcomes	N	Patients (TXA/non-TXA)	Overall effect		Heterogeneity	
			RR or MD (95% CI)	*P*	I^2^	*P*
Rate of blood transfusion						
All included studies	5	319/313	0.34 [0.14, 0.79]	0.02	0%	0.42
Randomized controlled trials	3	136/131	0.23 [0.07, 0.77]	0.01	NA	NA
RTSA	4	141/150	0.28 [0.10, 0.83]	0.02	0%	0.47
Estimated total blood loss (ml)						
All included studies	3	157/170	-249.24 [−338.74, −159.74]	< 0.00001	20%	0.29
Randomized controlled trials	2	80/76	− 357.92 [− 504.25, −211.59]	< 0.00001	0%	0.87
RTSA	2	95/101	−249.15 [−426.60, −71.70]	0.006	64%	0.10
Hb change within 48 h after surgery (g/dl)^a^						
All included studies	5	316/310	−0.64 [− 0.81, − 0.46]	0.009	0%	0.72
Randomized controlled trials	2	109/104	−0.65 [− 1.14, − 0.16]	< 0.00001	42%	0.19
RTSA	4	153/158	−0.64 [− 0.86, − 0.42]	< 0.0001	0%	0.66
IV TXA	5	260/255	−0.60 [− 0.79, − 0.41]	< 0.0001	0%	0.79
Topical TXA	1	56/55	−0.90 [− 1.42, − 0.38]	0.0007	NA	NA
Blood loss via drainage within 48 h after surgery (ml)^a^						
All included studies	4	160/155	−95.41 [−139.86, − 50.96]	< 0.001	65%	0.04
Randomized controlled trials	3	136/131	−105.78 [− 159.88, −51.68]	0.001	71%	0.03
RTSA	3	111/106	−84.56 [−145.72, −23.39]	0.007	80%	0.007
IV TXA	3	104/100	−110.04 [− 159.03, −61.06]	< 0.0001	65%	0.04
Topical TXA	1	56/55	−60.00 [−103.29, − 16.71]	0.007	NA	NA
Operation time (min)	3	183/161	−1.08 [−4.91, 2.74]	0.58	0%	0.42
Hospital stay (day)	2	159/137	− 0.04 [− 0.45, 0.37]	0.84	75%	0.05
Overall complications	6	343/337	0.44 [0.07, 2.96]	0.40	0%	0.95
Thromboembolic events	6	343/337	0.31 [0.01, 7.40]	0.47	NA	NA

*CI* Confidence interval; *Hb* Hemoglobin; *MD* Mean difference; *N* Number of studies; *NA* Not applicable; *RTSA* Reverse total shoulder arthroplasty; *TXA* Tranexamic acid

^a^Data from Gillespie 2015 and Friedman 2017 were estimated from median and range

### Allogenic blood transfusion rate

**Fig. 3:**
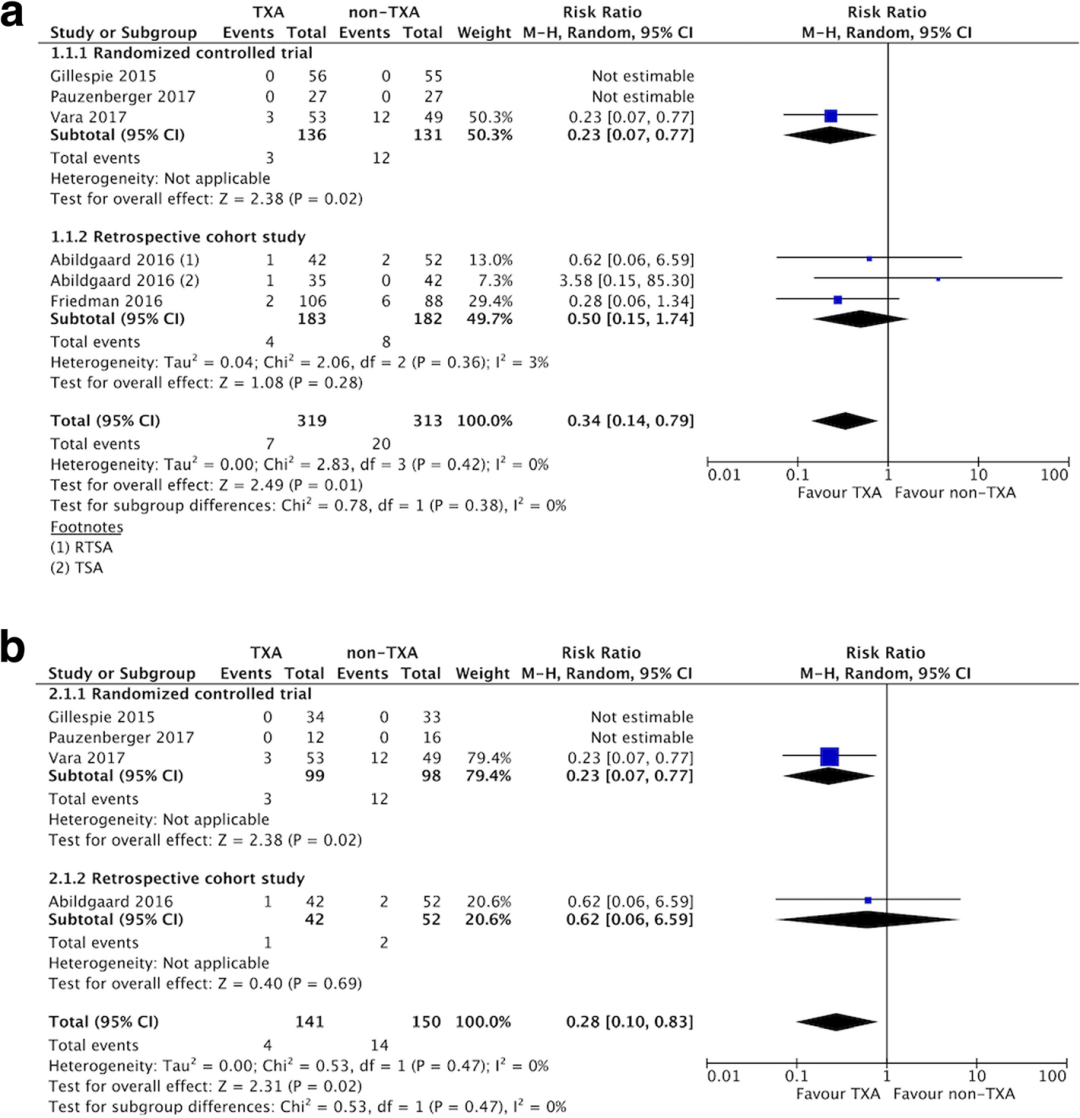
Forest plot and meta-analysis of the rate of blood transfusion. **a** All included studies (**b**) RTSA group

Five included studies [19, 20, 23, 33, 34] reported the blood transfusion rate in the TXA and non-TXA groups. The risk of the need for a blood transfusion was lower in the TXA group compared to the non-TXA group (7/319 vs. 20/313, RR 0.34, 95% CI 0.14–0.79; *P* = 0.01; *I*^2^ = 0%; Fig. 3a).

### Estimated total blood loss

**Fig. 4:**
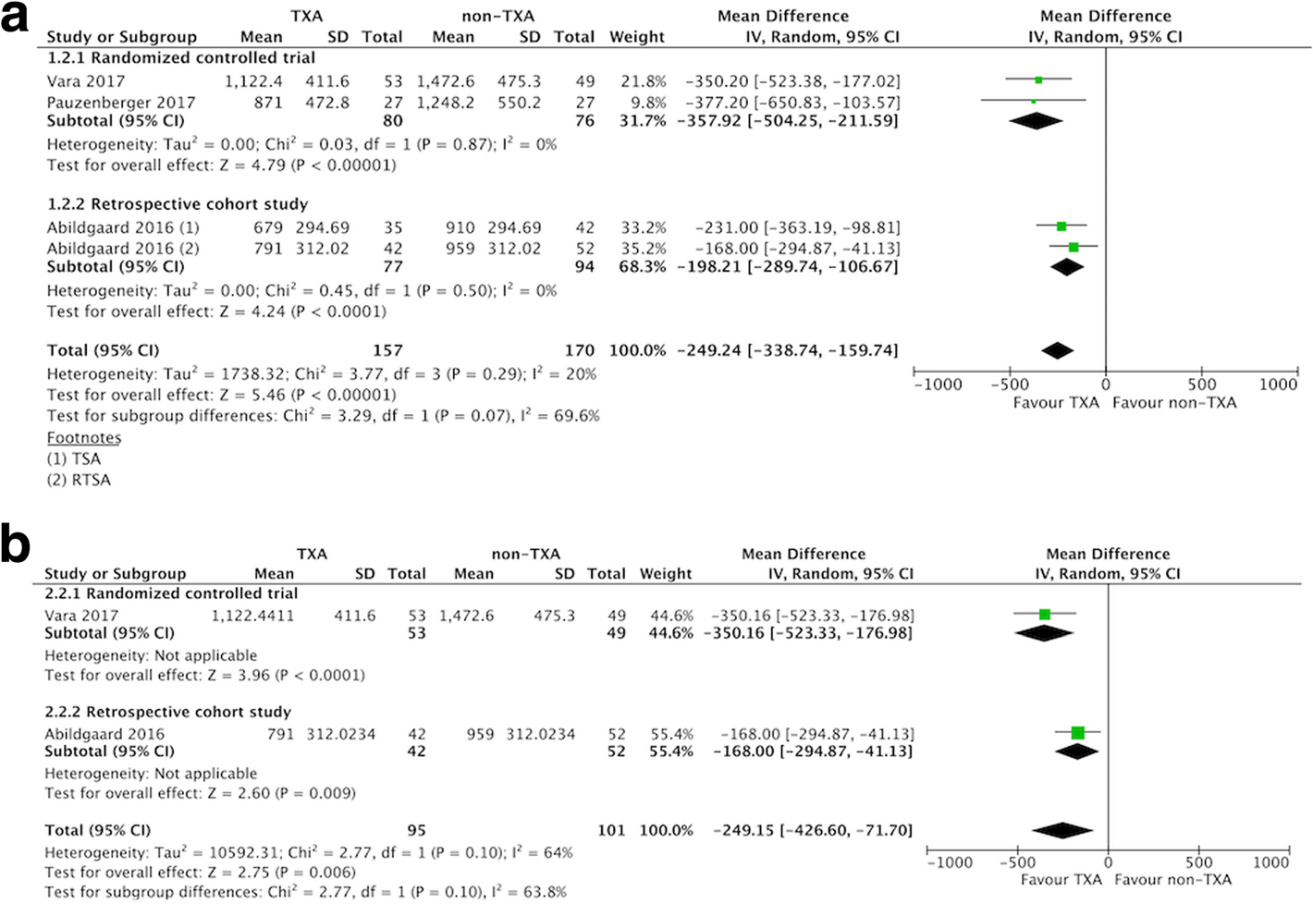
Forest plot and meta-analysis of estimated total blood loss. **a** All included studies (**b**) RTSA group

Of the included studies, three compared estimated blood loss in the two groups (157 patients in the TXA group vs. 170 patients in the non-TXA group) [23, 33, 34]. The pooled MD for estimated blood loss was − 249.24 ml (95% CI -338.74 to − 159.74 ml; *P* <  0.00001; *I*^2^ = 20%; Fig. 4a), indicating that the postoperative estimated blood loss was 249.24 ml lower in the TXA group compared to the non-TXA group.

### Changes in Hb (within 48 h after surgery)

**Fig. 5:**
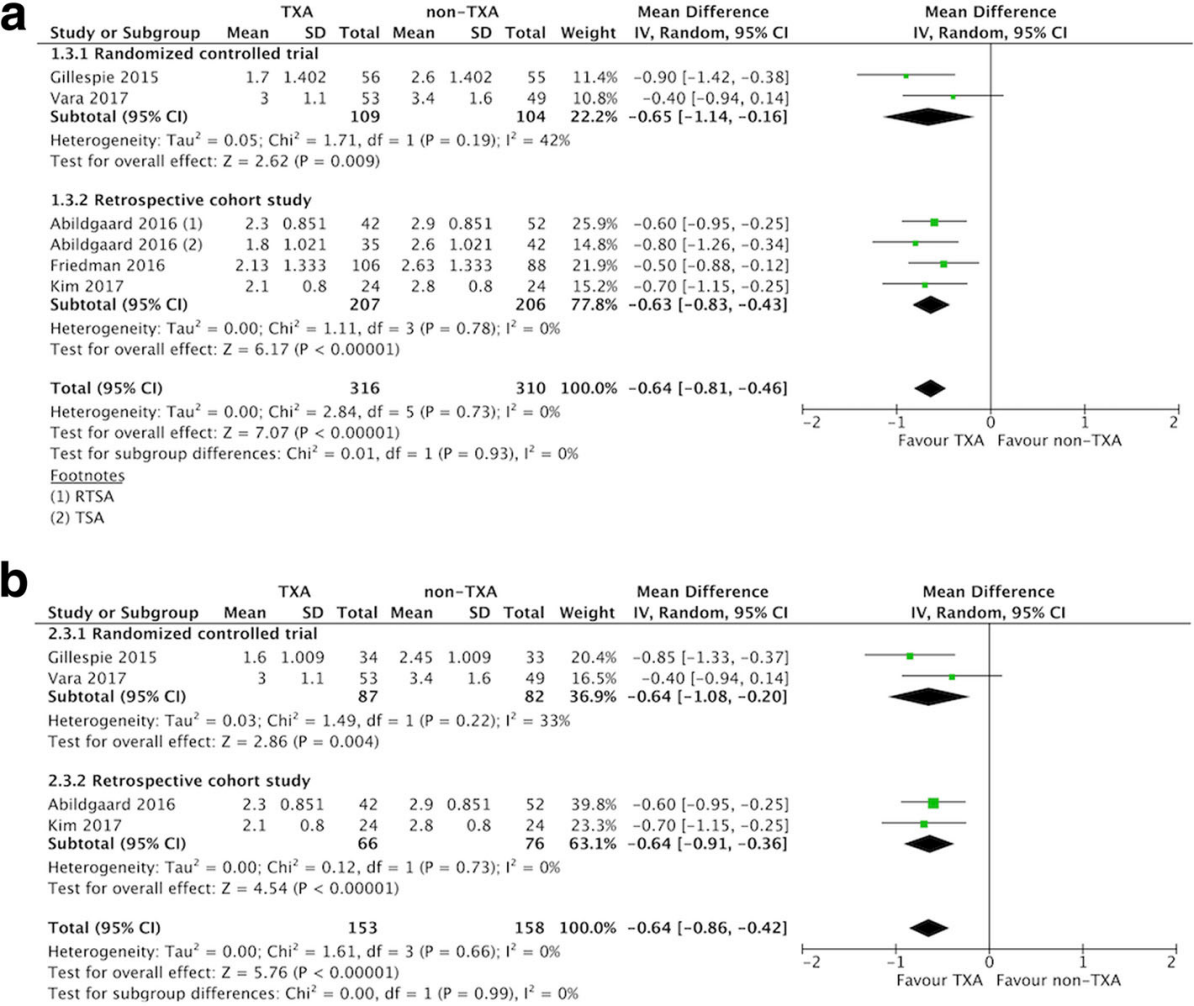
Forest plot and meta-analysis of Hb change. **a** All included studies (**b**) RTSA group (data from Gillespie 2015 were estimated from median and range)

Five studies reported changes in Hb within 48 h after surgery [19, 20, 24, 33, 34], with a pooled MD of − 0.64 g/dl (95% CI -0.81 to − 0.46 g/dl; *P* <  0.00001; *I*^2^ = 0%; Fig. 5a). This indicated that the postoperative decrease in Hb was 0.64 g/dl lower in the TXA group compared to the non-TXA group.

### Blood loss via drainage (within 48 h after surgery)

**Fig. 6:**
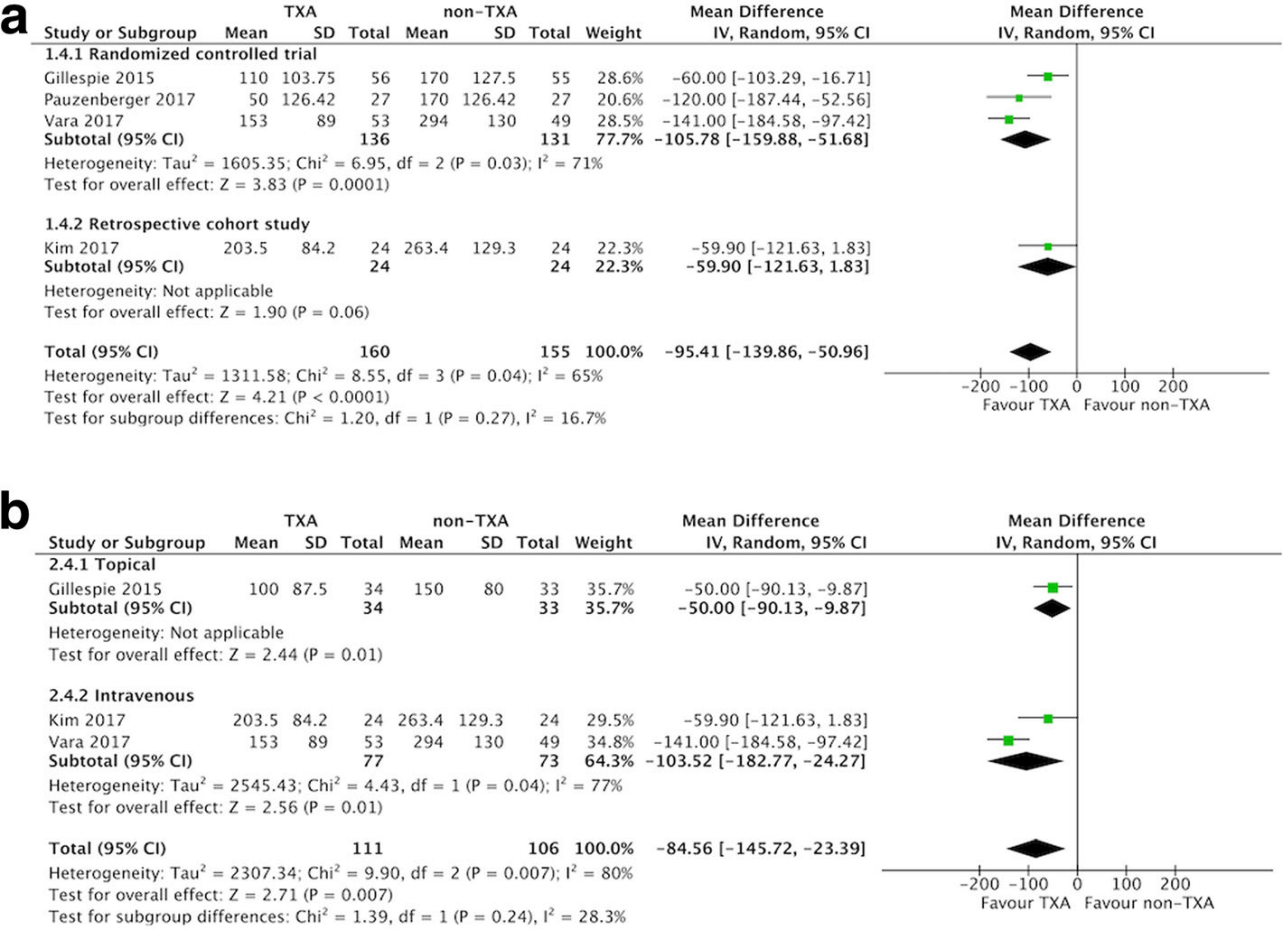
Forest plot and meta-analysis of blood loss via drainage. **a** All included studies (**b**) RTSA group (data from Gillespie 2015 and Friedman 2017 were estimated from median and range)

Three RCTs [20, 23, 34] and one RCS [24] compared blood loss in drainage in the TXA and the non-TXA groups, which consisted of 160 and 155 patients, respectively. The pooled MD of blood loss via drainage was − 95.41 ml (95% CI -139.86 to − 50.96 ml; *P* = 0.04; *I*^2^ = 65%; Fig. 6a), indicating that postoperative blood loss via drainage was 95.41 ml lower in the TXA group within 48 h after surgery.

### Operative time and hospital stay

**Fig. 7:**
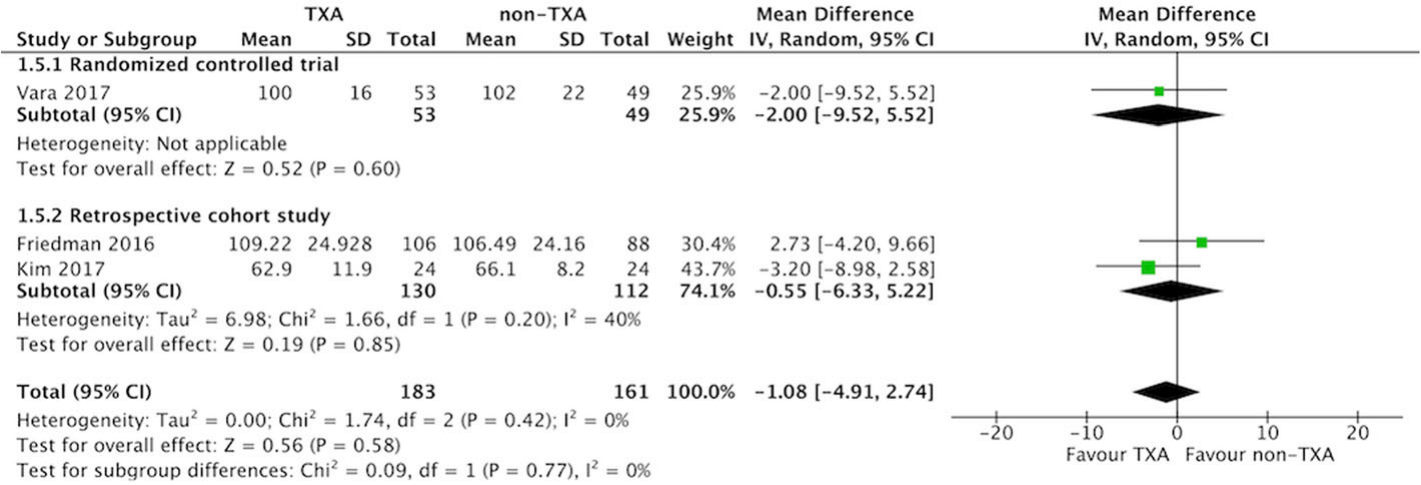
Forest plot of and meta-analysis of operative time

**Fig. 8:**
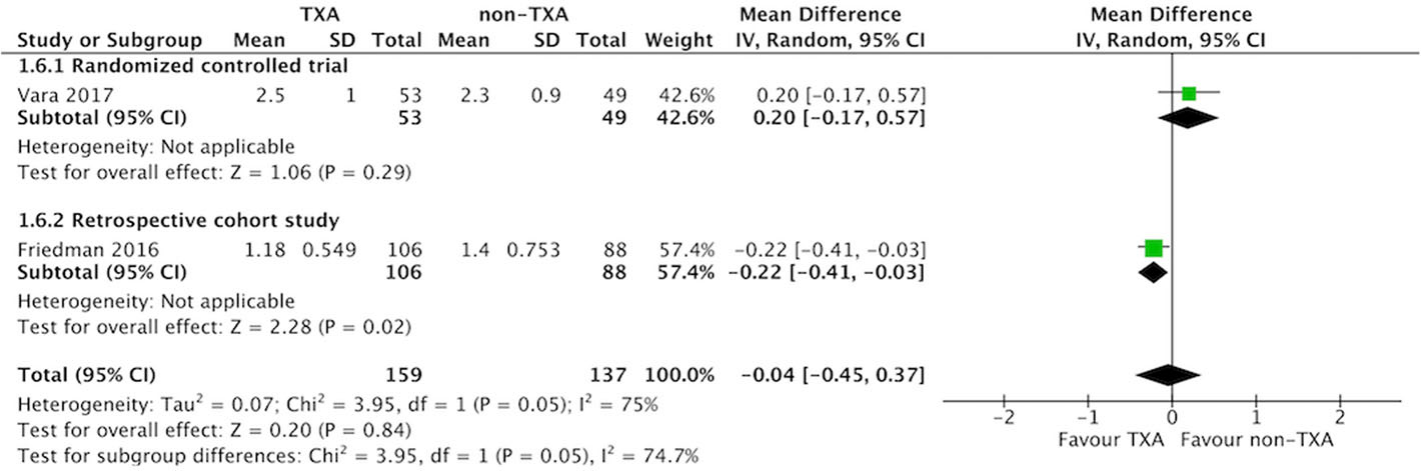
Forest plot of and meta-analysis of hospital stay

Of the included studies, three compared operative time between the two groups [19, 24, 34]. The pooled data showed that there was no significant difference between the two groups (MD − 1.08 min, 95% CI -4.91 to 2.74 min; *P* = 0.58; *I*^2^ = 0%; Fig. 7). Two studies compared the hospital stay between the two groups [19, 34], and the pooled data indicated that there was no significant difference regarding hospital stay between the two groups (MD − 0.04 days, 95% CI -0.45 to 0.37 days; *P* = 0.84; *I*^2^ = 75%; Fig. 8).

### Overall complications and thromboembolic events

**Fig. 9:**
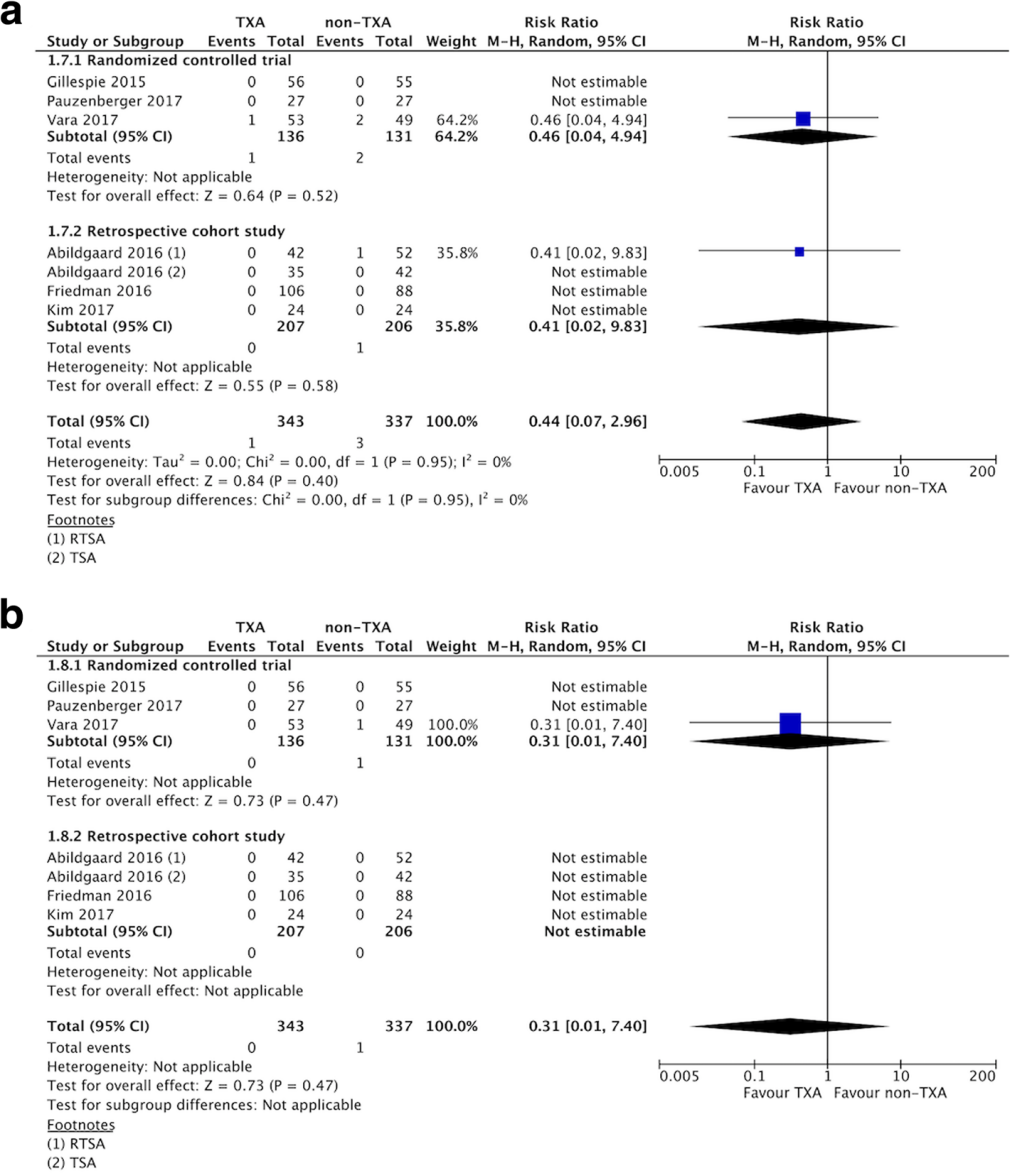
**a** Forest plot and meta-analysis of overall complications. **b** Forest plot and meta-analysis of thromboembolic complications

All six included studies reported data on the proportion of patients who developed complications and thromboembolic events during the study period. There were no significant differences in overall complications or thromboembolic events between the TXA and the non-TXA groups (overall complications: 1/343 vs. 3/337, RR 0.44, 95% CI 0.07 to 2.96; *P* = 0.95.; *I*^2^ = 0%; Fig. 9a; thromboembolic events: 0/343 vs. 1/337, RR 0.31, 95% CI 0.01 to 7.40; *P* = 0.47; Fig. 9b).

### Subgroup analysis

Subgroup analysis only including the three RCTs [20, 23, 34] did not affect the direction of effects of any of the outcomes including allogeneic blood transfusion rate, estimated total blood loss, change in postoperative Hb, and overall complications (Table 3). For data limited to RTSA, the TXA group had a lower allogenic blood transfusion rate, less estimated total blood loss, less change in Hb, and less blood loss via drainage compared with the non-TXA group (Table 3, Fig. 3-6b).

Considering the route of TXA administration, one study examined the topical use of TXA [20], and the other four studies examined the IV administration of TXA [19, 23, 24, 33, 34]. Regardless of whether topical or IV administration was used, the TXA group had less change in Hb than the non-TXA group, and there was no difference between the two routes of administration (subgroup difference, *P* = 0.27; Table 3, see Additional file 2: Figure S1). Four studies reported blood loss via drainage [20, 23, 24, 34]. The TXA group had less blood loss compared with the non-TXA group in both topical and IV subgroups (MD − 60.00 ml, 95% CI -103.29 to − 16.71 ml, *P* = 0.007; MD -110.04 ml, 95% CI -159.03 to − 61.06 ml, *P* <  0.0001, respectively; Table 3, see Additional file 3: Figure S2). Comparing these two subgroups, IV TXA was as effective as topical TXA in reducing blood loss via drainage (subgroup difference, *P* = 0.13, Table 3, see Additional file 3: Figure S2).

## Discussion

The main findings of this study are that the use of TXA in shoulder arthroplasty can reduce blood loss parameters including allogeneic blood transfusion rate, estimated total blood loss, change in Hb level, and blood loss via drainage without increasing the operative time, hospital stay, or the incidence of perioperative complications.

RTSA has been reported to be an independent predictor of the need for a blood transfusion after shoulder arthroplasty [2], which implied that patients undergoing RTSA bled more than TSA. Both the reverse design of implant geometry and the lack of intact cuff contribute to greater potential dead space in RTSA, resulting in more bleeding [24]. Our study shows that TXA reduces blood loss and the need for blood transfusion, suggesting that it could be applied to patients undergoing RTSA.

TXA has been shown to reverse the effect of plasminogen, thereby reducing blood loss and requirement for an allogeneic blood transfusion [35]. However, the most appropriate route of TXA administration is still under debate. One meta-analysis comparing the effectiveness and safety of IV versus topical administration of TXA in patients undergoing total knee arthroplasty showed that both IV and topical TXA had comparable efficacy in reducing blood loss and blood transfusion rates [36]. Another meta-analysis on total hip and knee arthroplasty also demonstrated similar results [37]. In the present study, both the IV and topical administration of TXA were effective in reducing blood loss and transfusion rates. For patients with a history of pulmonary embolism (PE) or deep vein thrombosis (DVT), the topical administration of TXA may be preferable.

Theoretically, TXA has a potential risk of thrombosis [14]. However, previous studies on total knee or total hip arthroplasty have not found an increased risk of thromboembolic events [16, 36–38]. Our findings suggest that TXA may not increase the risk of venous thromboembolic complications including PE and DVT in shoulder arthroplasty. This may be due to the use of allogeneic blood transfusion protocols with thromboprophylaxis including heparin, aspirin and mechanical prophylaxis in most of the included studies [23, 33, 34]. The low incidence of thromboembolic events may also be due to the routine use of chemical prophylaxis or the careful selection of patients with a higher risk of thromboembolism or the different nature between upper limb and lower limb surgery. In one study investigating the use of TXA in patients undergoing total hip arthroplasty without chemical prophylaxis for DVT, TXA was found to significantly increase the incidence of total DVT compared with the control group [39]. Since we found that both IV TXA and topical TXA were effective in reducing blood loss and transfusion rates, the topical administration of TXA may be preferable for patients with a history of PE or DVT.

To the best of our knowledge, this study is the most up-to-date systematic review and meta-analysis focusing on the use of TXA in shoulder arthroplasty. Compared to the previous systematic review, this study provides additional information about the efficacy of TXA with regards to the different types of shoulder arthroplasty and different routes of administration. This study also provides evidence supporting that TXA is a safe and efficient agent in reducing perioperative blood loss and transfusion rates without increasing complications, and that it can be applied in shoulder arthroplasty, especially for those at risk of requiring a blood transfusion.

This study has several important limitations. First, of the six studies, three were observational, with bias leading to inherent heterogeneity, even with high-quality scores. However, the subgroup analysis only including RCTs did not affect the direction of effects of any of the outcomes. In addition, we identified two ongoing trials from trial registries (NCT02569658, NCT01937559), and the findings of the present study may be different after including the results of these two trials. Second, the current study provides information on the route of TXA administration. However, this study can only provide indirect evidence about the comparison between the efficacy of IV TXA and topical TXA. Further trials with direct comparisons between IV TXA and topical TXA are needed to validate this finding. Finally, this study cannot make any conclusions on the optimal dose of TXA. Further head-to-head trials comparing different doses of TXA in shoulder arthroplasty are required to determine the optimal dose. Other limitations included non-standardized doses of TXA, variable postoperative chemoprophylaxis, and heterogeneous transfusion protocols which may have a significant effect on one of the main study endpoints.

## Conclusions

When used in shoulder arthroplasty, TXA is safe and effective in reducing perioperative blood loss and the need for a blood transfusion without increasing complications including thromboembolic events. TXA can be included as part of a comprehensive strategy for shoulder arthroplasty, especially for patients at a high risk of requiring a blood transfusion.

## Additional files

Additional file 1: **Appendix 1.** Database search strategy. (DOC 211 kb)

Additional file 2: **Figure S1.** Forest plot and meta-analysis of Hb change. The TXA group had a lower change in Hb than the non-TXA group in both the topical TXA and IV TXA subgroups. There was no significant subgroup difference. (data from Gillespie 2015 and Friedman 2017 were estimated from median and range). (TIFF 1923 kb)

Additional file 3: **Figure S2.** Forest plot and meta-analysis of blood loss via drainage. The TXA group had less blood loss via drainage than the non-TXA group in both topical TXA and IV TXA subgroups. There was no significant subgroup difference. (data from Gillespie 2015 and Friedman 2017 were estimated from median and range). (TIFF 1562 kb)

## Abbreviations

CI: Confidence interval
CTA: Cuff tear arthropathy
DP: Deltopectoral approach
ETBL: Estimated total blood loss
F: Female
Hb: Hemoglobin
Hct: Hematocrit
IV: Intravenous
M: Male
MD: Mean difference
N: Number of studies
N/S: Not shown
NA: Not applicable
NS: Normal saline
OA: Osteoarthritis
ON: Osteonecrosis
RA: Rheumatoid arthritis
RTSA: Reverse total shoulder arthroplasty
TSA: Total shoulder arthroplasty
TXA: Tranexamic acid
