# Supplementary material for: Tranexamic acid in total shoulder arthroplasty and reverse shoulder arthroplasty: a systematic review and meta-analysis
Source: BMC Musculoskelet Disord. 2018 Feb 17;19:60. doi: 10.1186/s12891-018-1972-3 (PMC5816518; doi:10.1186/s12891-018-1972-3)
Supplement: Supplementary file 1 — Appendix 1. Database search strategy. (DOC 211 kb) [file 12891_2018_1972_MOESM1_ESM.doc]

**Appendix 1. Database Search Strategy**

1. **Cochrane CENTRAL Search Strategy**

#1 MeSH descriptor: [Arthroplasty, Replacement] explode all trees

#2 ((joint* or arthroplasty or arthritic or shoulder) near replac*) or (joint* and replac* and (surg* or procedur* or operat*))

#3 #1 or #2

#4 MeSH descriptor: [Shoulder Joint] explode all trees

#5 shoulder

#6 #4 or #5

#7 #3 and #6

#8 total shoulder replacement OR total shoulder arthroplasty

#9 ((arthroplast* OR joint prosthe*) AND shoulder*):ti

#10 ("Shoulder Prosthesis" OR ((shoulder OR shoulder OR shoulder*) AND (replace* OR replacement OR replacing OR replaced OR arthroplast* OR arthroplasty OR arthroplastic OR prosthe* OR prosthesis OR prosthetic OR endoprosthe* OR implant* OR implant OR implants OR implanted))):ti

#11 #7 or #8 or #9 or # 10

#12 MeSH descriptor: [Tranexamic Acid] explode all trees

#13 tranexamic acid or amchafibrin or anvitoff or cyclokapron or cyklokapron or exacyl or kabi 2161 or lysteda or spotof or t-amcha or tranhexamic acid or transamin or ugurol or xp12b

#14 #12 or #13

#15 #11 and #14

1. **PubMed Search Strategy**

#1 "Arthroplasty"[Mesh] OR "Joint Prosthesis”[Mesh] OR "Prostheses and Implants"[Mesh] OR arthroplast*[ti] OR joint prosthe*[ti]

#2 "Shoulder"[Mesh] OR "Shoulder Joint"[Mesh] OR shoulder[ti] OR Shoulders[ti]

#3 #1 AND #2

#4 "Arthroplasty, Replacement, Shoulder"[mesh] OR "Shoulder Prosthesis"[ti] OR ((shoulder[ti] OR shoulders[ti] OR shoulder*[ti]) AND (replace*[ti] OR replacement[ti] OR replacing[ti] OR replaced[ti] OR arthroplast*[ti] OR arthroplasty[ti] OR arthroplastic[ti] OR prosthe*[ti] OR prosthesis[ti] OR prosthetic[ti] OR endoprosthe*[ti] OR implant*[ti] OR implant[ti] OR implants[ti] OR implanted[ti]))

#5 (#3 OR #4)

#6 “Tranexamic Acid” [Mesh]

#7 tranexamic acid or amchafibrin or anvitoff or cyclokapron or cyklokapron or exacyl or kabi 2161 or lysteda or spotof or t-amcha or tranhexamic acid or transamin or ugurol or xp12b

#8 #6 or #7

#9 #5 and #8

1. **Embase Search Strategy**

#1 ’Arthroplasty’/exp OR ‘Joint Prosthesis’/exp OR ‘Prostheses and Orthoses’/exp OR arthroplast*.ti OR “joint prosthe*”:ti

#2 ‘Shoulder’/exp OR shoulder*:ti

#3 (#1 AND #2)

#4 ‘Shoulder Arthroplasty’/exp OR ‘Shoulder prosthesis’/exp OR ‘Shoulder Prosthesis’:ti OR (shoulder*:ti AND (replace* OR replacement OR replacing OR replaced OR arthroplast* OR arthroplasty OR arthroplastic OR prosthe* OR prosthesis OR prosthetic OR endoprosthe* OR implant* OR implant OR implants OR implanted):ti)

#5 #3 OR #4

#6 ‘tranexamic acid’/exp

#7 tranexamic acid or amchafibrin or anvitoff or cyclokapron or cyklokapron or exacyl or kabi 2161 or lysteda or spotof or t-amcha or tranhexamic acid or transamin or ugurol or xp12b

#8 #6 OR #7

#9 #5 AND #8
