# Supplementary figures and images for: Tranexamic acid in total shoulder arthroplasty and reverse shoulder arthroplasty: a systematic review and meta-analysis
Source: BMC Musculoskelet Disord. 2018 Feb 17;19:60. doi: 10.1186/s12891-018-1972-3 (PMC5816518; doi:10.1186/s12891-018-1972-3)

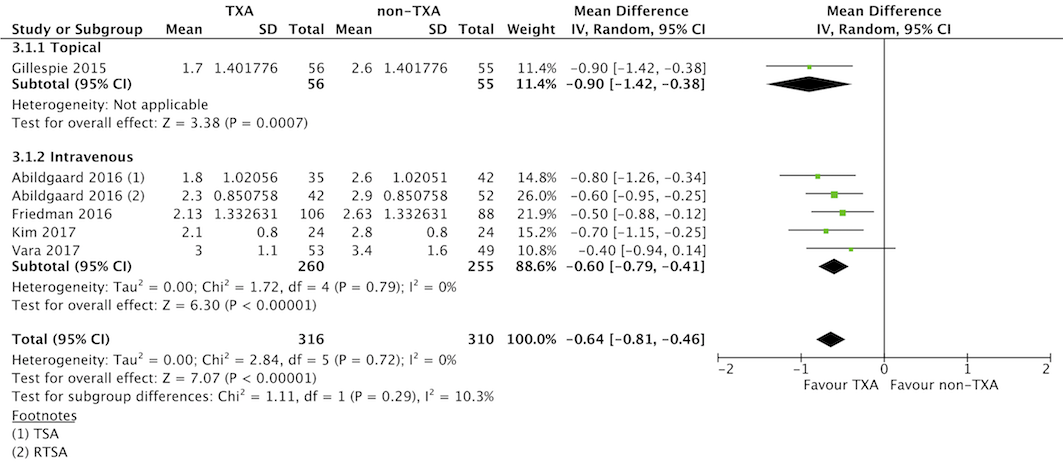

Supplement: Supplementary file 2 — Figure S1. Forest plot and meta-analysis of Hb change. The TXA group had a lower change in Hb than the non-TXA group in both the topical TXA and IV TXA subgroups. There was no significant subgroup difference. (data from Gillespie 2015 and Friedman 2017 were estimated from median and range). (TIFF 1923 kb) [file 12891_2018_1972_MOESM2_ESM.tiff]

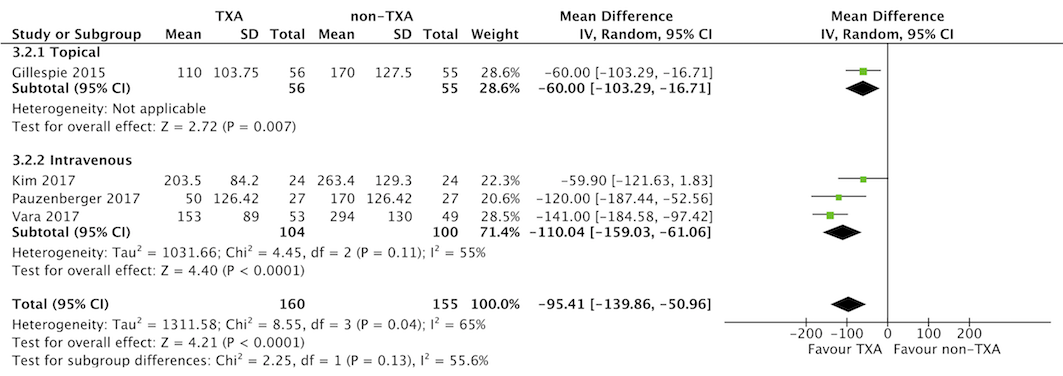

Supplement: Supplementary file 3 — Figure S2. Forest plot and meta-analysis of blood loss via drainage. The TXA group had less blood loss via drainage than the non-TXA group in both topical TXA and IV TXA subgroups. There was no significant subgroup difference. (data from Gillespie 2015 and Friedman 2017 were estimated from median and range). (TIFF 1562 kb) [file 12891_2018_1972_MOESM3_ESM.tiff]
